# Perfect health not so perfect after all – a methodological study on patient-reported outcome measures in 2574 patients following percutaneous coronary intervention

**DOI:** 10.1186/s12955-025-02360-4

**Published:** 2025-04-05

**Authors:** T. M. Norekvål, M. M. Iversen, K. Oterhals, H. Allore, B. Borregaard, T. R. Pettersen, D. R. Thompson, A. D. Zwisler, K. Breivik

**Affiliations:** 1 Centre on Patient-reported Outcomes, Department of Research and Development, Haukeland University Hospital, Jonas Lies vei 65, Bergen, 5021 Norway; 2 Department of Heart Disease, Haukeland University Hospital, Jonas Lies vei 65, Bergen, 5021 Norway; 3 Department of Clinical Science, University of Bergen, Laboratory Building, Haukeland University Hospital, Jonas Lies vei 87, Bergen, 5020 Norway; 4 Department of Health and Caring Sciences, Western Norway University of Applied Sciences, Inndalsveien 28, Bergen, 5063 Norway; 5 Department of Internal Medicine, Yale School of Medicine, 300 George St 7th FL, New Haven, CT 06437 USA; 6 Department of Biostatistics, Yale School of Public Health, 300 George St 7th FL, New Haven, CT 06437 USA; 7 Department of Cardiology, Odense University Hospital, J.B. Winsløws Vej 4, Odense C, 5000 Denmark; 8 Department of Clinical Research, University of Southern Denmark, J.B. Winsløws Vej 19, 3, Odense C, 5000 Denmark; 9 School of Nursing and Midwifery, Queen’s University Belfast, 97 Lisburn Rd, Belfast, BT9 7BL UK; 10 REHPA, The Danish Knowledge Centre for Rehabilitation and Palliative Care, Odense University Hospital, Vestergade 17, Nyborg, 5800 Denmark; 11 Regional Centre for Child and Youth Mental Health and Child Welfare, NORCE Norwegian Research Centre, P.O.B 22, Nygårdstangen, Bergen, 5838 Norway

**Keywords:** Patient-reported outcome measure, Disease-specific instrument, Generic instrument, Coronary artery disease, Percutaneous coronary intervention

## Abstract

**Background:**

Patient-reported outcome measures (PROMs) are crucial to capture patients’ health and illness status. Selecting the most suitable PROM to measure self-reported health in a specific study population is essential. Shortcomings of much used generic instruments have been identified in certain populations, and more investigation is needed to clarify the extent to which the generic instruments capture the aspects of health that really matter to patients. Therefore, the aim of this study was to determine floor and ceiling effects of a generic health utility instrument (EQ-5D-5L) in an international multi-centre cohort of patients after percutaneous coronary intervention (PCI) and further explore those with perfect health scores by using a disease-specific instrument.

**Methods:**

The CONCARD^PCI^ study was conducted at seven large referral PCI centres in Norway and Denmark between June 2017 and May 2020. In all, 2574 unique patients were available for this analysis. The generic EQ-5D-5L descriptive system and visual analogue scale, and the disease-specific Myocardial Infarction Dimensional Assessment Scale (MIDAS) comprising 35 items measuring seven areas of health status and daily life challenges were used to scrutinize the aims. Latent class analyses were conducted to identify classes with similar patterns of daily life challenges based on MIDAS item scores within the group of patients with best possible EQ-5D-5L score (‘perfect scorers’).

**Results:**

There was a large ceiling effect on the EQ-5D-5L score in patients with coronary artery disease (CAD) with 32% scoring the best possible EQ-5D-5L score, suggesting perfect health. Latent class analysis on the MIDAS revealed, however, four classes where 17-46% of the perfect scorers did perceive challenges in health, particularly related to symptoms of fatigue, and worries about risk factors and side effects of medication.

**Conclusion:**

To obtain an accurate picture of patients’ health status, these results emphasize that both generic and disease-specific patient-reported outcomes measures are needed to capture the distinct problems that patients with CAD experience after PCI. Caution should be made when using the EQ-5D-5L as the sole measure, particularly in priority settings, due to its potential ceiling effect and the fact that important aspects of patient health may be neglected.

**Trial registration:**

NCT03810612.

**Supplementary Information:**

The online version contains supplementary material available at 10.1186/s12955-025-02360-4.

## Introduction

Patient-reported outcome measures (PROMs) are crucial to capture patients’ health and illness status and its impact on their daily lives. Selecting the most suitable PROM to measure the construct of interest in a specific study population is essential [1–4]. Generic PROMs have the advantage that measurements are comparable between different patient groups. However, generic PROMs are less specific and sensitive to capture the distinctive problems that patients with a specific health condition experience compared to disease-specific PROMs [4, 5]. The EQ-5D is a frequently used generic multi-attribute health utility instrument [6]. In the original version, five health dimensions are measured at three levels (EQ-5D-3L) [7]. Although widely used, an insufficient discriminative power has been shown [8–11]. The more recent version, the EQ-5D 5-Level (EQ-5D-5L), was developed to improve accuracy and precision [6, 12, 13]. Still, a large ceiling effect (highest possible score) has been shown [14], and it is reported that utilisation of the EQ-5D-5L in health status measurements raises inconsistencies in capturing attributes and changes in disease-specific patient populations [15]. Yet, others report clinically important differences in health status associated with multimorbidity to be of similar magnitude using both versions of the EQ-5D [16]. The EQ-5D-5L, as a generic measure, targets mainly broad, physical domains of health and may fail to capture psychosocial burden and limitations associated with coronary artery disease (CAD) specifically. Sensitivity can however vary by population and context. Therefore, PROMs must be tested in the intended population to ensure acceptable standards of reliability and validity [4].

Regarding content coverage, and sensitivity and responsiveness, the EQ-5D-5L may not fully capture certain health domains and symptoms in patients with CAD. In a recent study, 51% of a patient population with chronic conditions reported missing important aspects. Among 17 identified aspects, fatigue and loss of energy were the most commonly reported omissions [15]. In addition, shortcomings have been identified in certain populations, especially for patients with ‘mild’ conditions [17]. These limitations highlight the need for potential modifications to the EQ-5D-5L, such as the use of ‘bolt-ons’, additional dimensions designed to improve its comprehensiveness. Further research is needed to clarify the extent to which the EQ-5D-5L adequately captures health aspects that really matter for patients with CAD. This responsiveness can be operationalised by exploring how patients reporting the best health profile on EQ-5D-5L respond on a disease-specific instrument. There is a lack of robust studies from Scandinavia using the EQ-5D-5L in CAD populations, and studies often include small populations, and/or no disease-specific instruments [5, 18, 19].

Therefore, the aim of this paper was to determine ceiling effects of a generic health utility instrument (EQ-5D-5L) in an international multi-centre cohort of patients after percutaneous coronary intervention (PCI) and further explore those with perfect health scores by using a disease-specific instrument.

## Methods

### Study design and setting

This is a secondary analysis in CONCARD^PCI^ - a large-scale multicentre cohort study with serial prospective survey data collection, clinical data and register-based follow-up. Patient-reported data were collected at baseline and three follow-up time points during one year. Seven large referral PCI centres in Norway and Denmark participated including both the national hospitals of the two countries [20]. The PCI centres perform from 900 to > 2000 (mean 1668) PCI procedures annually, having 629 to 1400 (mean 943) beds, and are referral centres for coronary angiography and PCI for a total of 37 local hospitals. We followed the STROBE (Strengthening the Reporting of Observational Studies in Epidemiology) guidelines when reporting of the results.

#### Study population

Patients were eligible to participate if they were ≥ 18 years of age, had undergone PCI, provided informed consent during the in-hospital assessment and were living at home at the time of index hospitalisation. Patients were excluded if they did not speak Norwegian/Danish, were unable to fill in the questionnaires due to reduced capacities, had expected lifetime less than 1 year, were undergoing PCI without stent implementation, had PCI related to transcatheter aortic valve implantation or MitraClip examination, or were previously enrolled in CONCARD^PCI^ (readmission). Patients who were too clinically unstable to participate following PCI, who would otherwise be eligible, were re-assessed until discharge. Because the questionnaires were designed for self-assessment, patients who needed a complete proxy were deemed ineligible.

### Measurement

#### Baseline characteristics

Socio-demographic characteristics included age, sex, cohabitation status, educational level and smoking status. Clinical characteristics included clinical status at admission, cardiac diagnosis, clinical pathway (acute, sub-acute and planned), prior PCI, prior cardiac surgery, prior implanted device, and previous cardiac and medical comorbidities.

### Outcome measures

*The EQ-5D-5L* is a generic questionnaire consisting of a 5-dimension descriptive system and a thermometer-like visual analogue scale ranging from 0 (worst health state) to 100 (best health state); the EQ VAS. The questionnaire comprises five dimensions, each describing one dimension (Mobility, Self-Care, Usual Activities, Pain/Discomfort, and Anxiety/Depression) with ratings on five levels of perceived health problems at the day they filled out the questionnaire, from no problems (1) to extreme problems (5). Level scores are presented as global health indices with a weighted total value for health status [21]. The lowest possible score represents the most severe health state, and the 11111-profile full health. In this paper, full health is labelled the term perfect health.

*The Myocardial Infarction Dimensional Assessment Scale (MIDAS)* is a disease-specific instrument comprising 35 items measuring seven areas of health status and daily life challenges during the last week: physical activity (12), insecurity (9), emotional reaction (4), dependency (3), diet (3), concerns regarding medication (2) and side effects (2). A 5-point ‘0–4’ Likert scale is used as the response set. Each subscale is transformed to range from 0 to 100, with higher scores indicating poorer health status on the measured dimension [22].

### Data collection

All patients undergoing PCI at seven large PCI centres were prospectively screened for eligibility. The screening was performed in the hospital setting by the site coordinator and trained CONCARD^PCI^ study nurses. Daily admission records and the operating programme were reviewed to identify potentially eligible patients. Electronic medical records were reviewed to confirm eligibility according to the inclusion and exclusion criteria, and to gather baseline characteristics for those included (T0). Patients were followed-up at 2 (T1), 6 (T2) and 12 (T3) months post-discharge. Patient self-report was collected either electronically using a tablet via a SurveyXact-link (SurveyXact version 12.9) or by paper, as preferred by the patient. Trained study nurses entered paper version data into the database. Non-responders received one reminder. Vital status was identified to avoid sending questionnaires to deceased patients or their family. Questionnaire packages were discussed with patient representatives and piloted at every measuring time point (T0-T3) before being employed in the large-scale cohort study. For this methodological study, we used data from baseline and T1. The study sample consisted of 2574 individuals (1481 from Norway and 1093 from Denmark) who had completed the EQ-5D-5L at T1 (Supplemental Fig. 1).

### Data analysis

Descriptive statistics and correlations were conducted using IBM SPSS Statistics for Windows, Version 26.0.0.1 (Released 2016. IBM SPSS Statistics for Windows, Version 26. Armonk, NY: IBM Corp). Latent class analyses (LCA) were conducted using the R-package ‘poLCA’, Version 1.4.1. LCA is a statistical method, similar to cluster analysis, which identifies hidden subgroups (latent classes) within a population based in shared patterns in individuals’ responses to observed indicators. LCA was used to identify similar patterns of daily life challenges based on MIDAS item scores, referred to as classes, among participants who reported that they had experienced symptoms or daily life challenges during the last week. Item response categories 2–4 (sometimes, often and always) were collapsed into one category (sometimes or more) due to a low cell count on the two highest responses on most items (Supplemental Table 1). As LCA is a pure statistical method, it weights all of the items equally, and thus does not weight them differently due to differing clinical significance, for instance. One to seven class solutions were analysed of which each solution was tested using 200 random starting values to avoid local solutions. The solution was considered stable if the best likelihood was replicated at least four times. Choice of which solution to retain was based on several information criteria including the Bayesian Information Criterion (BIC) as primary [23], but also Adjusted BIC (ABIC), Akaike Information Criterion (AIC) and Consistent AIC (CAIC). After information criteria were compared, inspection of profile plots regarding parsimony was used to decide on the LCA model that differ in shape and not only level of symptoms or daily life challenges [24]. A LCA 4 class analysis of which country (Danish versus Norwegian) served as a predictor of latent class membership was also performed with multinominal regression analyses using class 4 (the largest and healthiest group) as a reference group.

### Ethics approval and consent to participate

The ethical guidelines of the World Medical Association, Declaration of Helsinki (2008) and the legislation in Norway and Denmark guided the study. At inclusion, a detailed letter informed the potential participant of the study, and the right to withdraw from the study at any time without any reason was underlined. All patients provided informed consent to participate. Data are kept in strict confidence in locked files at research servers to protect the participants’ privacy. Approval by the Norwegian Regional Committee for Ethics in Medical Research in Western Norway was granted (REK 2015/57), and from the Data Protection Agency in the Zealand region for the Danish centres (REG-145-2017). CONCARD^PCI^ is registered at clinicaltrials.gov (NCT03810612) on January 18th 2019.

### Patient and public involvement

Two patient representatives with a history of CAD, who had been trained as patient representatives in health care and research settings, were included from the start of the project and involved in setting the research question and outcome measures. After publication, they will be involved in the dissemination of the project results.

## Results

The typical patient was male (78%), aged 66 years, cohabiting (77%), non-smoker (83%) with no higher education (72%). Patients had several comorbid conditions, most commonly hypertension (51%) or hypercholesterolemia (47%). Indication for PCI was typically stable CAD (30%), NSTEMI (27%) and STEMI (21%) using arteria radialis (79%) as access route (Table 1).

**Table 1:** Patient characteristics of study sample at baseline in the CONCARD^PCI^ study (*N* = 2574)

Characteristics n* (%)	
**Sex**	
Men	2026 (78.7)
Women	548 (21.3)
**Age at admission**,** mean ± SD (range)**	66.0 **±** 10.38 (30–96)
**Living alone** **Not living alone**	525 (21.8) 1888 (78.2)
**Education**	
Primary school	473 (19.1)
Vocational school	1075 (43.5)
Upper secondary school	225 (9.1)
University college or university, < 4 years	388 (15.7)
University college or university, ≥ 4 years	312 (12.6)
**Smoking status**	
Never smoker	747 (30.1)
Former smoker	1369 (55.2)
Current smoker	364 (14.7)
**Indication for PCI**	
Stable coronary artery disease	790 (30.0)
Unstable angina	343 (13.0)
NSTEMI	713 (27.1)
STEMI	561 (21.3)
Other	226 (8.6)
**Access route for PCI**	
Arteria radialis	2033 (79.0)
Arteria femoralis	438 (17.3)
Arteria radialis and arteria femoralis	77 (2.6)
**Previous PCI**	632 (24.7)
**Previous CABG**	234 (9.1)
**Other cardiac surgery**	62 (2.4)
**Previously implanted device**	87 (3.4)
Pacemaker	38 (2.6)
ICD	20 (1.4)
CRT-P	2 (0.1)
CRT-D	7 (0.5)
**Previous cardiovascular comorbidities**	
Atrial fibrillation/flutter	314 (12.3)
Cerebrovascular disease	145 (5.7)
Coronary artery disease	847 (33.1)
Chronic heart failure	192 (8.0)
Hypercholesterolemia	1194 (46.8)
Hypertension	1310 (51.2)
Myocardial infarction	506 (19.8)
Peripheral artery disease	131 (5.1)
**Previous medical comorbidities**	
Anxiety and depression	226 (8.9)
- Under treatment anxiety and depression	151 (5.9)
Arthritis	144 (5.6)
Arthrosis	192 (7.5)
Asthma	144 (5.7)
Chronic inflammatory bowel disease	54 (2.1)
Chronic renal failure	102 (4.0)
Chronic skin conditions	97 (3.8)
Cancer	290 (11.4)
- Under treatment cancer	80 (3.1)
Chronic obstructive pulmonary disease	170 (6.7)
Diabetes (insulin)	137 (5.4)
Diabetes (tablets)	330 (12.9)
Neurological disease	81 (3.2)
Osteoporosis	113 (4.4)
Other chronic conditions	565 (22.4)
Other muscle-skeletal conditions	188 (7.5)
Other psychological conditions	54 (2.1)
- Under treatment other psychological conditions	38 (1.5)

*Some variables have some missing (*n* = 2227–2574)

Abbreviations: CABG: coronary artery bypass grafting; CRT-D: cardiac resynchronization therapy with defibrillator; CRT-P: cardiac resynchronization therapy with pacemaker; ICD: implantable cardioverter defibrillator; PCI: percutaneous coronary intervention; SD: standard deviation

### Ceiling effect in EQ-5D-5L

**Fig. 1:**
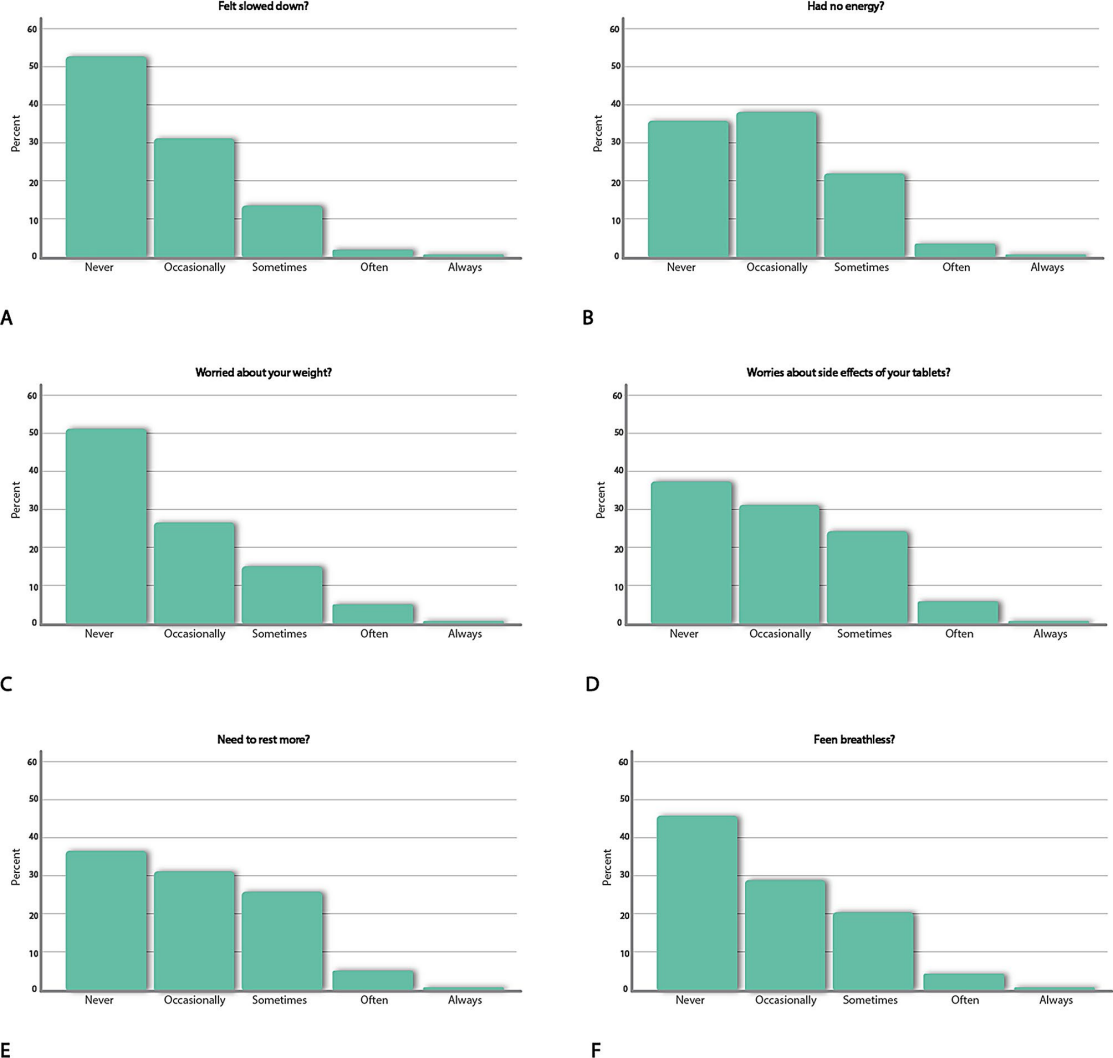
Response from perfect scorers on the EQ-5D-5 L to single items on the disease-specific MIDAS in the CONCARD^PCI^ study (*n* = 815)

While there was none of the patients who reported worst possible health, there was a strong ceiling effect on the EQ-5D-5L in patients with CAD as 32% had the lowest possible score suggesting perfect health (Supplemental Fig. 2). This ceiling effect was nearly identical in both countries; for Norway (31.6%, *n* = 468) and Denmark (31.7%, *n* = 347). In contrast, only 1.7% (*n* = 40) reported the best health state on MIDAS. Although almost one third of patients with CAD displayed a perfect score on the generic EQ-5D-5L, Fig. 1 shows that perfect scorers reported challenges in several disease specific items.

**Fig. 2:**
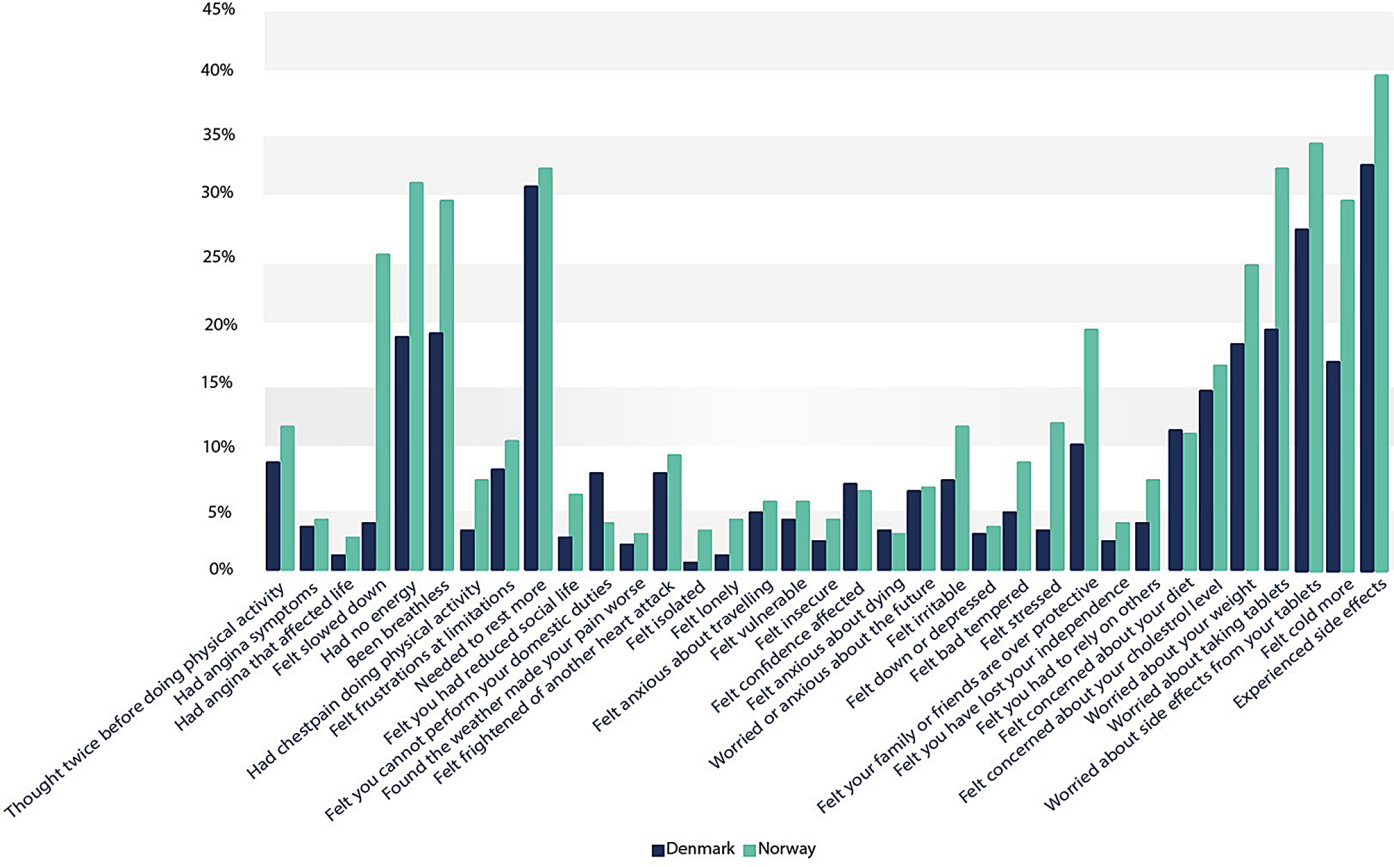
Proportion of perfect EQ-5D-5L scorers who reported challenges on specific MIDAS items (sometimes or more often during the last week) in the CONCARD^PCI^ study (*n* = 760)

The proportion of individuals with perfect scores on the EQ-5D-5L, but who endorsed category 2–4 (sometimes or more often during the last week), on the disease-specific MIDAS items are presented in Fig. 2.

Supplemental Table 1 displays a complete frequency table. Besides for Norwegian perfect scorers being in general more likely to affirm these higher response categories, the largest proportions were tied to physical activity items measuring fatigue, worries about risk factors and side effects, and experiencing side effects. In Norway, the proportion ranged from 25 to 40% among perfect scorers on these items, while they, except for one item, ranged from 18 to 33% for their Danish counterparts. Only 4% of the Danes reported that they ‘Felt slowed down’ sometimes or more often during the last week. However, we ran a LCA 4 class analysis of which country (Danish versus Norwegian) served as a predictor of latent class membership. Using Class 4 (the largest and healthiest group) as a reference group, multinominal regression analyses revealed that country did not statistically significantly predict membership in any of the classes.

### EQ VAS in the perfect scorers

While most of the perfect scorers rated their general health as 80 out of 100 or above, 28% rated their health as lower than this (Supplemental Fig. 3).

To explore whether the MIDAS items were associated with this variance we correlated them with the EQ VAS score. Most of the MIDAS items had a weak (*r* <.10) and often non-significant correlation with the EQ VAS score in this group. An exception was items tapping fatigue of which the correlations were in the − 0.20 to − 0.30 range (‘Felt slowed down’, *r*=-.27; ‘Had no energy’, *r*=-.29; ‘Been breathless’, *r*=-.24; ‘Needed to rest more’, *r*=-.23). A few other items had a larger negative relation than *r*=-.15 (‘Felt frustrated at your limitations’, *r*=-.19; ‘Felt you cannot perform domestic duties’, *r*=-.18).

### Latent class analyses

For further inspection of the item responses of the perfect scorers’ group, we applied LCA on the MIDAS items. We retained the four-class solution as this had the best fit on two of the fit indices with the lowest BIC and CAIC value (Table 2) which was considered the most parsimonious solution when inspecting the profile plots.

**Table 2:** Information criteria for seven class solutions from latent class analyses in the CONCARD^PCI^ study

	Log-likelihood	df	BIC	AIC	ABIC	CAIC	Entropy
Model 1	-20179.91	690	40824.14	40499.81	40601.86	40894.14	NaN
Model 2	-18018.97	619	36973.25	36319.95	36525.51	37114.25	0.921
Model 3	-17459.04	548	36324.34	35342.07	35651.15	36536.34	0.898
Model 4	-17146.26	477	36169.74	34858.52	35271.10	36452.74	0.901
Model 5	-16934.41	406	36217.01	34576.81	35092.91	36571.01	0.887
Model 6	-16764.11	335	36347.39	34378.23	34997.83	36772.39	0.802
Model 7	-16611.20	264	36512.52	34214.39	34937.51	37008.52	0.838

BIC: Bayesian Information Criterion; ABIC: Adjusted BIC; AIC: Akaike Information Criterion; CAIC: Consistent AIC

**Fig. 3:**
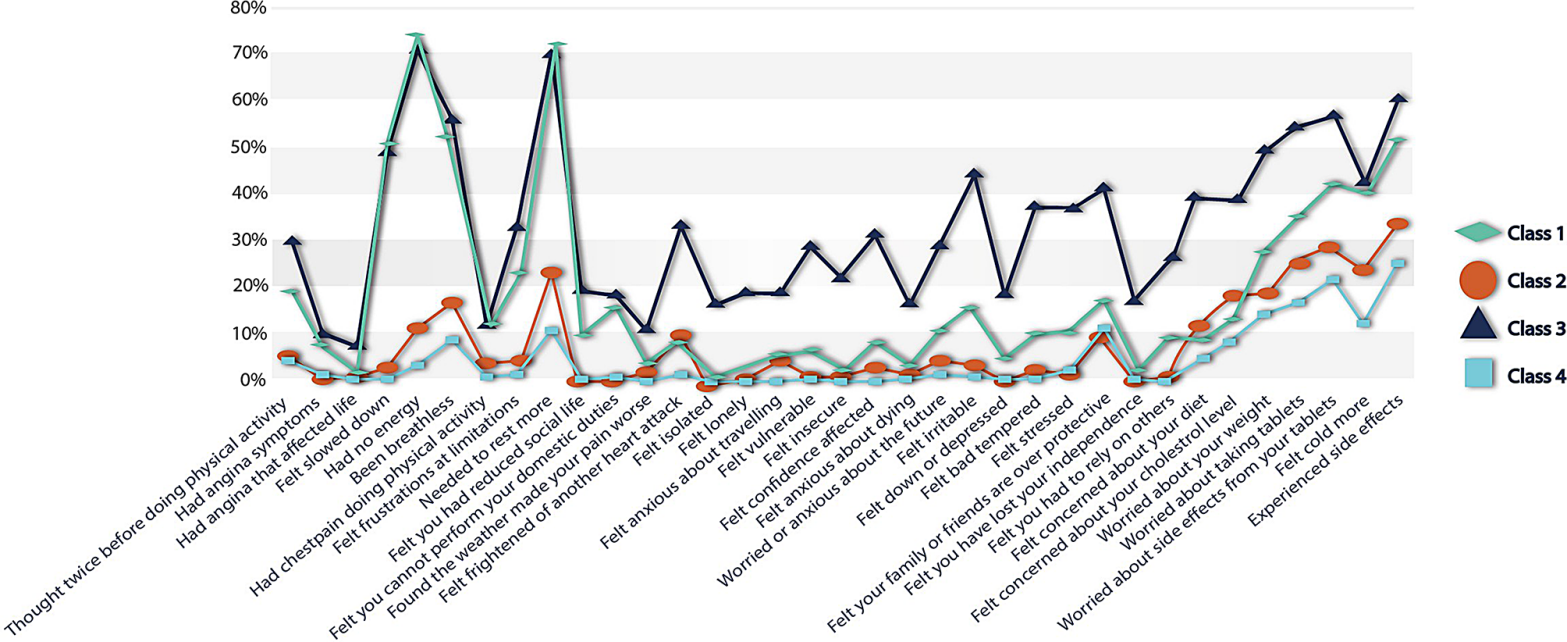
Proportion of perfect EQ-5D-5L scorers who have experienced challenges in MIDAS items sometimes or more during the last week in the CONCARD^PCI^ study (*n* = 760). ‘Major fatigue and side effects’ (Class 1) = 17%, ‘Only some fatigue and side effects’ (Class 2) = 25%, ‘Poor cardiac quality of life’ (Class 3) = 12%, and ‘Good cardiac quality of life’ (Class 4) = 46%

The four classes revealed were labeled as ‘Major fatigue and side effects’ (Class 1), ‘Only some fatigue and side effects’ (Class 2), ‘Poor cardiac quality of life’ (Class 3), and ‘Good cardiac quality of life’ (Class 4) (Fig. 3).

The smallest group (‘Poor cardiac quality of life’), estimated to be 12% of the sample, was most likely to have experienced challenges across MIDAS items at least sometimes during the last week. The ‘Good cardiac quality of life’ group (46%) comprised a very low proportion of individuals (< 10%) reporting challenges sometimes or more during the last week. There was a tendency across profiles that the challenges were more pronounced for items tapping symptoms of fatigue as well as experiencing and worrying about side effects of medication. The ‘Major fatigue and side effects’ participants in Class 1 (17%) experienced fatigue symptoms at least sometimes during the last week (50–70%) and worried about side effects. The ‘Only some fatigue and side effects’ participants (25%) in Class 2 comprised individuals tapping fatigue as well as experiencing and worrying about side effects. There were some exceptions, the most noteworthy was the high proportion of individuals in ‘Major fatigue and side effects’ (50–70%) who reported that they had experienced fatigue symptoms. This proportion was nearly identical to what was the case for ‘Poor cardiac quality of life’, even though this class had a considerably lower proportion who reported challenges on most other items. Supplemental Fig. 4 depicts a summary of the main results of the study.

## Discussion

This large representative study demonstrates a strong ceiling effect on the EQ-5D-5L score in patients with CAD two months after PCI. Among those scoring the best possible score, and thus suggesting perfect health, the disease-specific MIDAS questionnaire revealed that this perfect score group perceived challenges in health. Symptoms related to experiencing fatigue at least sometimes in the last week, and worries about risk factors and side effects of medication were most prominent. In addition, fatigue was associated with lower perceived global health measured by the EQ VAS score.

A recent systematic review recommended rigorous exploration of the responsiveness of the EQ-5D-5L since large proportions of respondents reporting the best health profile were observed for general population studies as well as patient populations, although less for the latter [6]. In our study, we explored more in-depth the substantial ceiling effect in measurements of the EQ-5D-5L in a large multi-centre patient population with CAD. The disease-specific MIDAS questionnaire revealed that the EQ-5D-5L perfect score group did report challenges in health. This was confirmed across LCA profiles, as there was a tendency for the proportion to be largest for items tapping fatigue as well as experiencing and worrying about side effects. These symptoms are in line with the clinical picture in these patients [25] and are supporting the plausibility of low sensitivity of the EQ-5D-5L for this patient population [26]. Fatigue is found to increase the risk of recurrent cardiac events in patients undergoing PCI [25, 27]. Further, it is associated with poorer quality of life in patients with CAD [28] and thereby important to identify in patients after PCI. Worries about side effects of medication was confirmed in interviews with patients undergoing first-time PCI [29]. Patients faced multiple, interacting challenges in trying to adhere to prescribed medications following discharge, and experienced symptoms of fatigue. This underlines that both generic and disease-specific PROMs are needed to capture the distinct problems that patients with CAD or other specific health conditions experience [4].

The EQ-5D was conceptualised to measure deviations from full health (or negative health) and is more prone to larger ceiling effects than instruments that include positive health dimensions (e.g., the SF-6D) [5]. A study found that while 47% reported no limitations on all EQ-5D dimensions (i.e. perfect scorers), only 5.8% did so on the SF-6D. Even though both the EQ-5D and SF-6D are generic they are measuring different domains. For example, EQ-5D-5L includes a specific domain for self-care, which is not explicitly covered in SF-6D. Conversely, SF-6D includes domains for social functioning and vitality, which are not explicitly covered in EQ-5D-5L. The SF-6D is derived from the SF-36 health survey and includes more detailed questions, potentially offering greater sensitivity in certain areas. Still, the SF-6D did not discriminate between perfect scores with different morbidities as did the SF-12 [10]. However, it has been suggested to overcome shortcomings and improve accuracy and precision of the EQ-5D-5L by adding dimensions, which are often referred to as bolt-ons [30]. Our findings inform improvements to PROMs such as adding bolt-ons to the EQ-5D-5L. Incorporating targeted bolt-ons could more accurately reflect patients’ symptom burden and guide more effective clinical decision-making and patient care.

A systematic review summarizing bolt-on dimensions that were tested on the EQ-5D-5L, concluded that some studies showed that possible bolt-on dimensions had added value in a descriptive or valuation sense. To standardise wording and response options, evaluations of minimal important gain and development of quality assessment guidelines are needed [30].

Utilisation of the EQ-5D-5L in health status measurements raises inconsistencies in capturing quality of life attributes and changes in disease-specific patient populations [15]. In our study, fatigue items were correlated with perceived global health measured by the EQ VAS score among the perfect scorers group. Symptoms suggesting fatigue (‘Felt slowed down’, ‘Had no energy’, ‘Been breathless’, ‘Needed to rest more’, ‘Felt frustrated at your limitations’) are significantly related to the illness trajectory of this patient group and of clinical importance. Our results are supported by other published data, which strengthen our suggestion that the EQ-5D-5L does not capture some aspects of health that really matter for patients after PCI. Although sensitivity can vary by population and context, understanding which factors may reduce health outcomes is important for the implementation of more efficient disease management programmes [31]. Moreover, as EQ-5D-5L scores are used in health economic evaluations and priority settings in the healthcare system, important indicators of patient-reported health can be neglected [32]. It has been pointed out that calculating pooled mean estimates in modelling of cardiovascular disease-related outcomes in economic evaluations can be useful [32]. However, the usefulness of these estimates in priority settings in the healthcare system can be questioned, when not capturing important correlates associated to the illness trajectory. Policymakers should consider incorporating disease-specific measures like MIDAS or using bolt-ons to ensure a more accurate representation of patient health and treatment benefits.

In this multicenter study, almost one out of three patients after PCI reported perfect health as measured by the EQ-5D-5L. We found the same trends in the results for participants from both countries, supporting replicability, which is a central tenet of the scientific method underlining the robustness of the data [26]. However, the results showed that fatigue-related challenges were more frequently reported in Norwegian than Danish participants. This emphasise the importance of testing our research question in two countries.

The LCA profiles underline the importance of tapping fatigue and worries about side effects of medications. This is important knowledge for healthcare personnel. Consequently, this need to be emphasised when counselling patients after PCI. Moreover, to promote a systematic focus on these issues, disease-specific questionnaires such as the MIDAS could be useful as screening tools in clinical practice [33], leading to more recognition of fatigue and worries about risk factors and side effects of medications. To further support the clinical significance of our findings, it is however important to replicate them in other populations to give further empirical support for practical implications in treatment.

### Methodological issues

Data were collected at baseline and two-month follow-up to allow for symptoms and daily life challenges to be measured after some time post-discharge. The study had an adequate sample size to avoid random errors substantially influencing the results. The inclusion rate was high (82%) as 3430 out of 4209 eligible patients were included in the study. Although non-participants may still represent a limitation, the age and sex distribution in our study are consistent with national data provided by the Norwegian Registry on Invasive Cardiology and the Danish Heart Registry. Further, the response rate at two-month follow-up was high (81%), leaving 2574 patients for analysis in this methodological study. As for the specific questionnaires under study, the number of patients not responding to any items in the EQ-5D-5L or the MIDAS were similar (*n* = 92 versus *n* = 94). Of those responding to the questionnaires, 85% (*n* = 2340) had complete responses on the MIDAS and 93% (*n* = 2574) had complete responses to the EQ-5D-5L. Missing data was handled by listwise deletion. Given the fact that as much as 93% (760 out of 815) of the perfect scorers had complete data on the MIDAS, the potential bias is probably negligible. This methodological work is based on secondary analysis of data from a large cohort study. In studies with methodological purposes, the limitation of secondary analyses on Type I error is however lessened. Excluding certain patients for clinical reasons may have limited the broader generalizability of the findings although the inclusion criteria were wide. Despite a vast age representation we did not stratify for age. However, our analyses showed the same tendency for those under and above 80 years as both groups had highest score on symptoms of fatigue, worries about risk factors and side effects of medication. A final limitation is that the recall period of the EQ-5D-5L and the MIDAS is slightly different. While the EQ-5D asks about the respondents health today, the MIDAS asks about the last week, which could potentially influence how patients perceive and report on their health states. While the EQ-5D-5L captures a more immediate assessment, the MIDAS’s longer timeframe might capture episodic fluctuations in CAD symptoms, potentially leading to variations in reported outcomes. Consequently, direct comparisons of results across the two instruments should consider the impact of these different recall windows on patient responses.

## Conclusion

There was a strong ceiling effect on the EQ-5D-5L score in patients with CAD two months following discharge. Among those scoring the best possible score, and thus suggesting perfect health, the disease-specific MIDAS revealed that this perfect score group did however perceive challenges in health. LCA profiles confirmed a tendency for the proportion to be largest for items tapping fatigue as well as experiencing and worrying about side effects of medication. These symptom areas are significantly related to the illness trajectory for this patient group and therefore of clinical importance. The results on the EQ-5D-5L regarding ceiling effect cannot however be generalized to all generic questionnaires and requires further investigation. Still, to receive an accurate picture of patients’ health, these results emphasise that both generic and disease-specific PROMs are needed to capture the distinct problems that patients with a specific health condition experience. Future research should further investigate the integration of generic and disease-specific PROMs in longitudinal studies to assess their predictive value for long-term health outcomes in patients with CAD. This could lead to better tools for evaluating treatment effectiveness and guiding continuous improvement in cardiovascular care.

## Electronic supplementary material

Below is the link to the electronic supplementary material.

Supplementary Material 1

## Data Availability

The datasets generated and/or analysed during the current study are not publicly available according to the Norwegian data protection legislation. Analysis files (SPSS syntaxes, R scripts etc.) will be available from the corresponding author upon reasonable request.

## Abbreviations

ABIC: Adjusted BIC
AIC: Akaike Information Criterion
BIC: Bayesian Information Criterion
CAD: Coronary artery disease
CAIC: Consistent AIC
EQ-5D-5L: EQ-5D 5-Level
LCA: Latent class analyses
MIDAS: Myocardial Infarction Dimensional Assessment Scale
PCI: Percutaneous coronary intervention
PROMS: Patient-reported outcome measurements
