# Supplementary material for: Perfect health not so perfect after all – a methodological study on patient-reported outcome measures in 2574 patients following percutaneous coronary intervention
Source: Health Qual Life Outcomes. 2025 Apr 5;23:35. doi: 10.1186/s12955-025-02360-4 (PMC11972467; doi:10.1186/s12955-025-02360-4)
Supplement: Supplementary file 1 — Supplementary Material 1 [file 12955_2025_2360_MOESM1_ESM.pdf]

Supplemental Table 1. Frequency of perfect scorers on the generic EQ-5D-5L to each response category on single items on the disease-specific Myocardial Infarction Dimensional Assessment Scale (MIDAS).

**Following you heart attack, how often during the last week have you…**		**Never**	**Occasionally**	**Sometimes**	**Often**	**Always**
	**Country**	**n (%)**	**n (%)**	**n (%)**	**n (%)**	**n (%)**
Thought twice before you undertook physical activity (e.g. housework or going to the shops)?	D<br>N	248 (71.5)<br>298 (64.1)	69 (19.9)<br>113 (24.3)	20 (5.8)<br>44 (9.5)	7 (2.0)<br>10 (2.2)	3 (0.9)<br>0 (0.0)
Had angina symptoms (e.g. chest pain or tightness)?	D<br>N	265 (76.6)<br>382 (82.9)	69(19.9)<br>60 (13.0)	12 (3.5)<br>16 (3.5)	0 (0.0)<br>0 (0.0)	0 (0.0)<br>3 (0.7)
Had angina (chest pain or tightness) that affected your life?	D<br>N	303 (88.1)<br>414 (90.2)	37 (10.8)<br>33 (7.2)	3 (0.9)<br>8 (1.7)	0 (0.0)<br>0 (0.0)	1 (0.3)<br>4 (0.9)
Felt slowed down?	D<br>N	243 (70.2)<br>181 (39.0)	90 (26.0)<br>165 (35.6)	12 (3.5)<br>99 (21.3)	0 (0.0)<br>18 (3.9)	1 (0.3)<br>1 (0.2)
Had no energy?	D<br>N	147 (42.5)<br>143 (30.9)	134 (38.7)<br>176 (38.0)	58 (16.8)<br>121 (26.1)	5 (1.4)<br>22 (4.8)	2 (0.6)<br>1 (0.2)
Been breathless?	D<br>N	178 (51.6)<br>188 (40.6)	101 (29.3)<br>138 (29.8)	57 (16.5)<br>106 (22.9)	9 (2.6)<br>26 (5.6)	0 (0.0)<br>5 (1.1)
Had chest pain or tightness when undertaking physical activity?	D<br>N	280 (80.9)<br>358 (77.0)	55 (15.9)<br>73 (15.7)	10 (2.9)<br>29 (6.2)	0 (0.0)<br>2 (0.4)	1 (0.3)<br>3 (0.6)
Felt frustrated at your limitations?	D<br>N	259 (74.9)<br>318 (68.8)	59 (17.1)<br>95 (20.6)	25 (7.2)<br>40 (8.7)	2 (0.6)<br>9 (1.9)	1 (0.3)<br>0 (0.0)
Needed to rest more?	D<br>N	133 (38.4)<br>162 (35.0)	106 (30.6)<br>151 (32.6)	89 (25.7)<br>119 (25.7)	16 (4.6)<br>27 (5.8)	2 (0.6)<br>4 (0.9)
Felt you have a reduced social life?	D<br>N	288 (83.5)<br>369 (79.5)	47 (13.6)<br>67 (14.4)	9 (2.6)<br>25 (5.4)	0 (0.0)<br>1 (0.2)	1 (0.3)<br>2 (0.4)
Felt you cannot perform your domestic duties?	D<br>N	252 (72.8)<br>384 (82.4)	67 (19.4)<br>64 (13.7)	24 (6.9)<br>12 (2.6)	1 (0.3)<br>2 (0.4)	2 (0.6)<br>4 (0.9)
Found the weather made your pain worse?	D<br>N	315 (91.3)<br>409 (87.8)	22 (6.4)<br>43 (9.2)	7 (2.0)<br>12 (2.6)	0 (0.0)<br>2 (0.4)	1 (0.3)<br>0 (0.0)
Felt frightened you will have another heart attack?	D<br>N	234 (67.6)<br>314 (67.7)	85 (24.6)<br>107 (23.1)	25 (7.2)<br>41 (8.8)	1 (0.3)<br>2 (0.4)	1 (0.3)<br>0 (0.0)
Felt isolated?	D<br>N	324 (93.6)<br>418 (89.7)	19 (5.5)<br>33 (7.1)	2 (0.6)<br>13 (2.8)	0 (0.0)<br>1 (0.2)	1 (0.3)<br>1 (0.2)

Felt lonely?	D	322 (93.1)	20 (5.8)	3 (0.9)	0 (0.0)	1 (0.3)
	N	404 (87.1)	41 (8.8)	15 (3.2)	3 (0.6)	1 (0.2)
Felt anxious about travelling?	D	283 (82.0)	46 (13.3)	15 (4.3)	0 (0.0)	1 (0.3)
	N	359 (77.2)	80 (17.2)	25 (5.4)	1 (0.2)	0 (0.0)
Felt vulnerable?	D	278 (80.6)	53 (15.4)	13 (3.8)	0 (0.0)	1 (0.3)
	N	347 (74.6)	92 (19.8)	24 (5.2)	2 (0.4)	0 (0.0)
Felt insecure?	D	285 (82.4)	53 (15.3)	7 (2.0)	0 (0.0)	1 (0.3)
	N	351 (75.6)	94 (20.3)	17 (3.7)	1 (0.2)	1 (0.2)
Felt your confidence has been affected?	D	265 (76.6)	57 (16.5)	21 (6.1)	2 (0.6)	1 (0.3)
	N	351 (75.6)	83 (17.9)	28 (6.0)	1 (0.2)	1 (0.2)
Felt anxious about dying?	D	274 (79.2)	61 (17.6)	9 (2.6)	1 (0.3)	1 (0.3)
	N	404 (87.3)	45 (9.7)	13 (2.8)	1 (0.2)	0 (0.0)
Worried or felt anxious about the future?	D	245 (71.0)	78 (22.6)	19 (5.5)	2 (0.6)	1 (0.3)
	N	307 (65.9)	127 (27.3)	31 (6.7)	1 (0.2)	0 (0.0)
Felt irritable?	D	233 (67.3)	88 (25.4)	24 (6.9)	0 (0.0)	1 (0.3)
	N	260 (55.8)	152 (32.6)	51 (10.9)	3 (0.6)	0 (0.0)
Felt down or depressed?	D	282 (81.5)	54 (15.6)	9 (2.6)	0 (0.0)	1 (0.3)
	N	351 (75.6)	96 (20.7)	16 (3.4)	1 (0.2)	0 (0.0)
Felt bad tempered?	D	260 (75.1)	69 (19.9)	16 (4.6)	0 (0.0)	1 (0.3)
	N	273 (58.7)	151 (32.5)	40 (8.6)	1 (0.2)	0 (0.0)
Felt stressed?	D	257 (74.3)	78 (22.5)	10 (2.9)	0 (0.0)	1 (0.3)
	N	273 (59.0)	135 (29.2)	50 (10.8)	4 (0.9)	1 (0.2)
Felt your family or friends are over protective?	D	229 (66.4)	81 (23.5)	28 (8.1)	4 (1.2)	3 (0.9)
	N	260 (56.0)	114 (24.6)	79 (17.0)	10 (2.2)	1 (0.2)
Felt you have lost your independence?	D	320 (92.5)	18 (5.2)	7 (2.0)	0 (0.0)	1 (0.3)
	N	376 (81.2)	69 (14.9)	14 (3.0)	2 (0.4)	2 (0.4)
Felt you have to rely on others?	D	290 (83.8)	43 (12.4)	11 (3.2)	1 (0.3)	1 (0.3)
	N	341 (74.0)	86 (18.7)	31 (6.7)	1 (0.2)	2 (0.4)
Felt concerned about your diet?	D	238 (68.8)	68 (19.7)	35 (10.1)	4 (1.2)	1 (0.3)
	N	297 (64.0)	116 (25.0)	45 (9.7)	5 (1.1)	1 (0.2)
Felt concerned about your cholesterol level?	D	189 (54.8)	106 (30.7)	40 (11.6)	7 (2.0)	3 (0.9)
	N	257 (55.2)	132 (28.3)	63 (13.5)	10 (2.1)	4 (0.9)
Worried about your weight?	D	189 (54.6)	94 (27.2)	42 (12.1)	18 (5.2)	3 (0.9)
	N	224 (48.3)	126 (27.2)	84 (18.1)	25 (5.4)	5 (1.1)

Worried about taking tablets?	D<br>N	194 (56.1)<br>188 (40.3)	85 (24.6)<br>127 (27.3)	52 (15.0)<br>122 (26.2)	12 (3.5)<br>23 (4.9)	3 (0.9)<br>6 (1.3)
Worried about side effects from your tablets?	D<br>N	142 (41.0)<br>159 (34.3)	109 (31.5)<br>145 (31.3)	70 (20.2)<br>123 (26.6)	20 (5.8)<br>30 (6.5)	5 (1.4)<br>6 (1.3)
Felt the cold more?	D<br>N	241 (69.9)<br>229 (49.8)	46 (13.3)<br>95 (20.7)	40 (11.6)<br>97 (21.1)	16 (4.6)<br>34 (7.4)	2 (0.6)<br>5 (1.1)
Experienced side effects (e.g. cold hands or feet/ going to the toilet at night) from your medication?	D<br>N	151 (43.6)<br>182 (39.1)	82 (23.7)<br>99 (21.2)	73 (21.1)<br>121 (26.0)	34 (9.8)<br>55 (11.8)	6 (1.7)<br>9 (1.9)

Abbreviations: D: Denmark, N: Norway.

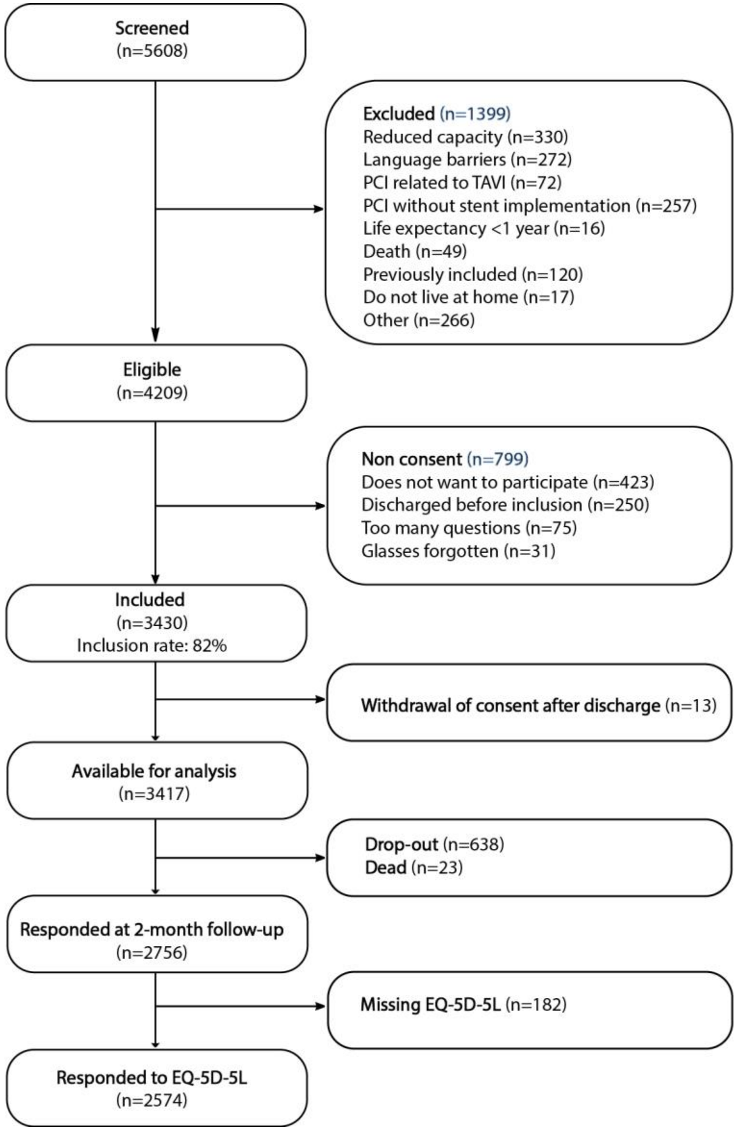

Supplemental Figure 1. Flowchart of inclusion of patients after percutanous coronary intervention in the CONCARD^{PCI} study (n=2574).

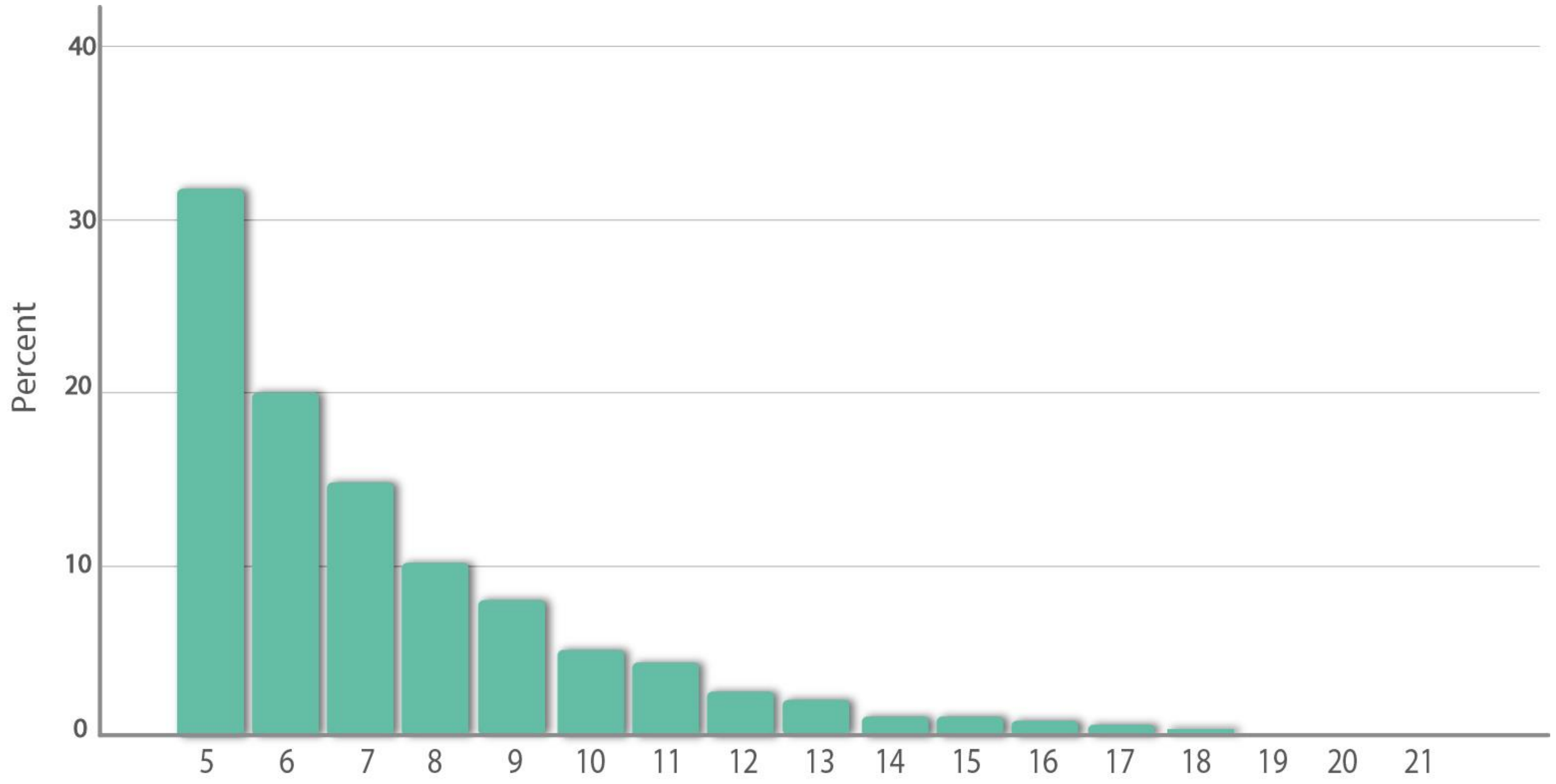

Supplemental Figure 2. Distribution of EQ-5D-5L total score in patients after percutanous coronary intervention in the CONCARD^{PCI} study (n=2574).

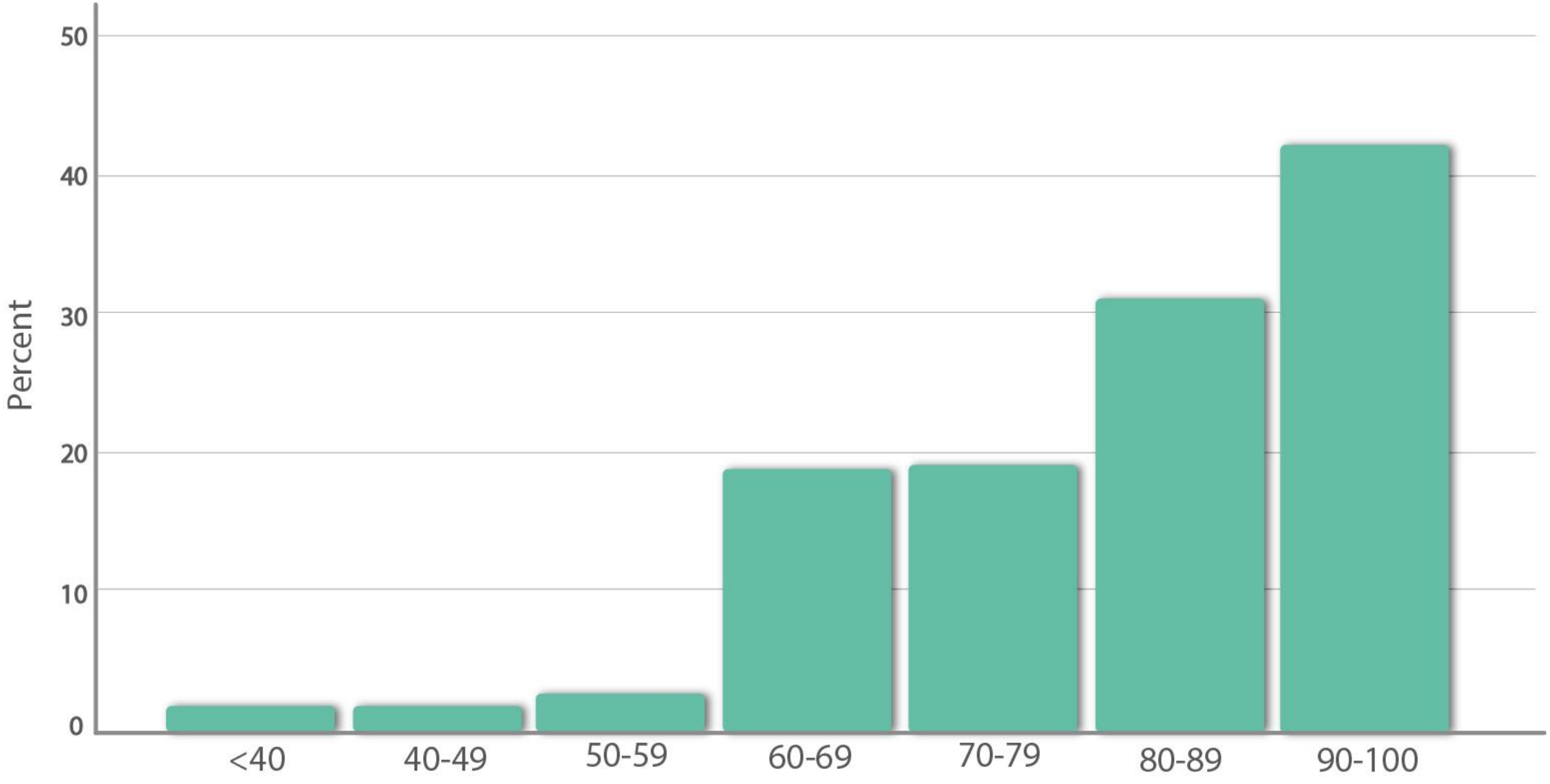

Supplemental Figure 3. Perfect scorers' rating on overall health (EQ-VAS) in the CONCARD^{PCI} study.

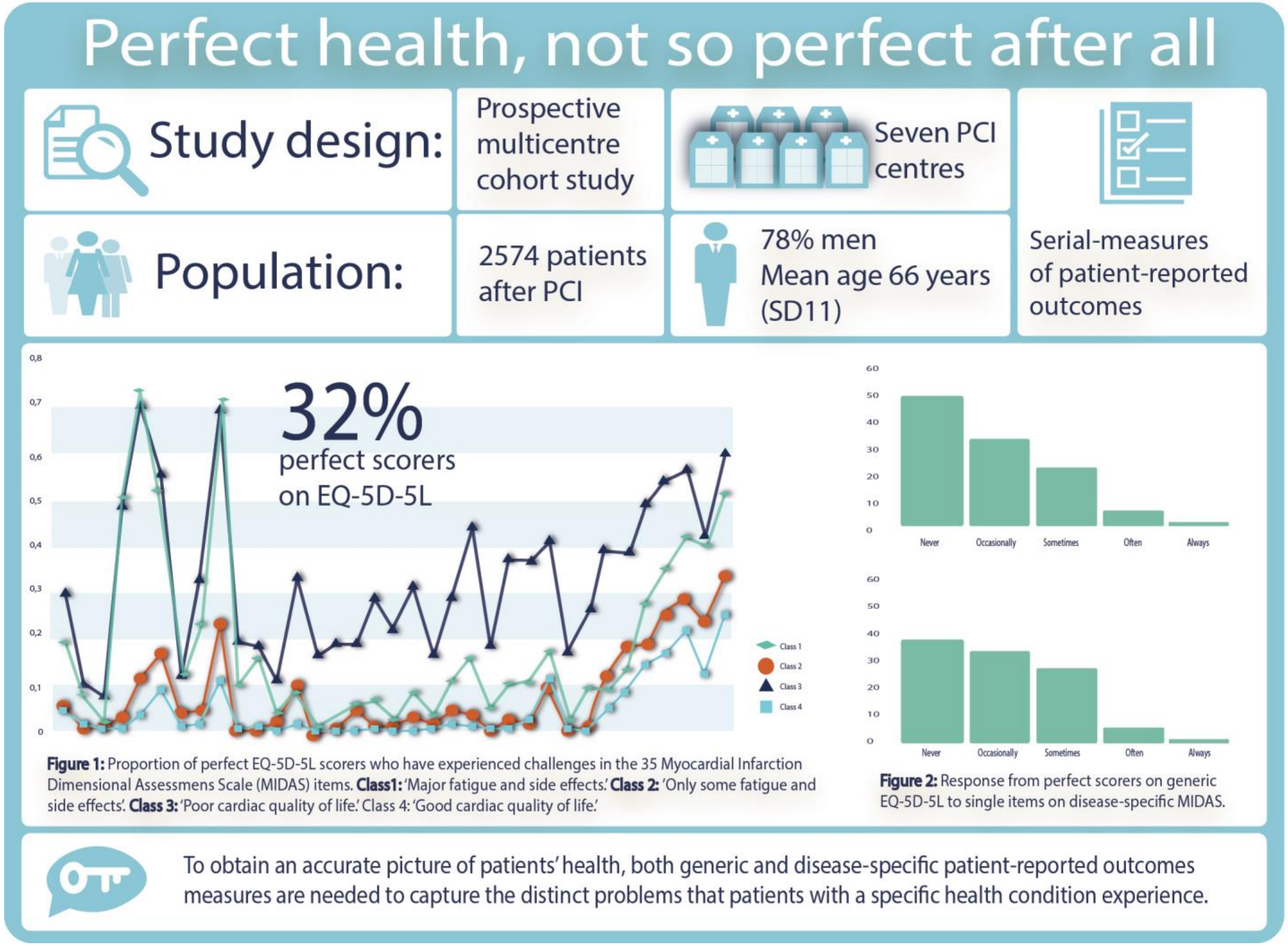

Supplemental Figure 4. Summary of the main results.
